# The Etiology of Chorea and Its Treatment

**Published:** 1902-02

**Authors:** 

**Affiliations:** Editor in Chief; Secretary of the Editorial Department of the *Journal de Médecine Interne*, Paris


### TRANSLATION.

### The Etiology of Chorea and Its Treatment.

By Dr. BESANÇON, Editor in Chief, and Dr. PAULESCO, Secretary of the Editorial Department of the *Journal de Médecine Interne*, Paris.

[Translated from *Journal de Médecine Interne*, April 1, 1901.]

THE absence of any positive ideas on the etiology and pathogenesis of chorea has distinctly manifested itself in the treatment of this affection. That the different remedies proposed against the disease have not given satisfactory results, is easily to be understood in view of the inadequacy of the theories which serve as the basis of their use. Not one of these theories is acceptable, since they do not take cognisance of the entire group of symptoms. The theory that chorea is a neurosis does not rest upon a firm foundation, for chorea does not present any of the characteristics of a neurosis. It is not hereditary; it can be cured easily and perfectly and it is not a life-long ailment. The same arguments apply to the theory that chorea is a process of degeneration.

The articular and endocardial symptoms so frequent in chorea have led it to be regarded as a simple manifestation of rheumatism. Under the name of rheumatism, however, are comprised several diverse conditions, some of which have been interpreted as of neuropathic and others of microbial origin. Chorea is evidently not of neuropathic nature, as is shown by the presence of endocarditis in some cases. On the other hand the articular manifestations of chorea differ from those of severe rheumatism, both in their clinical character and in their evolution. Chorea has been attributed to several special microbes, which have been described in detail, but whose specific character has not been proven. It has also been referred to an infection by ordinary bacteria, such as the staphylococcus, but its symptomatology does not correspond to that produced by these organisms.

Any one who takes account of all the conditions under which chorea develops, and the various affections from which it has its source, is led to consider this malady as of microbial origin, resembling rheumatic fever in its articular manifestations and in the presence of endocarditis, and, on the other hand, akin to whooping-cough and tetanus in the absence of fever and the existing motor phenomena.

As a matter of fact, chorea, like a majority of microbial maladies, is observed especially during childhood. Among 50 cases observed by us in the hospital during the last 14 years, 17 were aged less than 10 years; 29, 10 to 15 years, and 4 only between 15 and 18 years. Like acute rheumatism, chorea is an exception after the age of 20 years. While it has sometimes been observed in young pregnant females, 17 to 23 years old, it is chiefly met with in childhood.

Defective hygienic conditions furnish a distinct predisposing cause, its frequency being particularly great among children of the poorer classes. Insufficient nutrition, unhealthy dwelling places, impure air, moisture and want of sunlight, are among the principal factors which favor the development of chorea. Aside from these, it is more frequent during the cold season in the autumn and spring, and also often follows in the wake of febrile and exhausting diseases, such as eruptive fevers, pneumonia, and whooping-cough. Hence the same etiological conditions are concerned in its causation as in that of a number of germ diseases. Finally, the manifest augmentation of the volume of the pulse-rate demonstrated by us in six patients who have been under our observations is an argument in favor of this view.

Case I.—One of our patients, a little girl, suffered intensely with pains in the joints. We administered aspirin, a remedy which had given us excellent results in articular troubles, especially those of acute rheumatism. Three or four days later the pains had disappeared, but at the same time we noticed that the ataxic movements had completely ceased. This unexpected result did not fail to attract our attention, the more so as the salicylates, which we had employed on several occasions in chorea, had not afforded any material effects. The same experience was more or less repeated in the other cases.

The following observations will serve to give an idea of the almost specific action of the drug in chorea.

Case II., G. J., 11 years old. Parents addicted to alcohol. Bad hygienic conditions, living in a damp dwelling. Chorea had been present since two years, following a fright; three recurrences having taken place at intervals. The involuntary movements were of moderate intensity, walking being possible. There were present articular pains and endocarditis. Aspirin, 30 grains daily, was taken for eight days. On the fourth day, there was a distinct amelioration; the choreic movements diminished, and finally disappeared, the patient recovering her entire health.

Case III.—B. F., 10 years old; intense chorea; walking impossible. The patient received aspirin, 22 grains daily. Six days later the choreic movements had almost entirely ceased, speech becoming normal soon after.

Case IV., C. N., 11 years old; markedly severe chorea; the patient unable to stand. On admission to the hospital her body was covered with ecchymoses, following falls and blows. The treatment consisted of aspirin, 40 grains daily. At the end of two days there was considerable improvement. The ataxic movements were no longer present, although the speech remained difficult, and the expression of her face remained stupid. Ten days later the patient was discharged cured.

Case V.—B. E., 11 years old. Intense chorea; unable to speak. She received aspirin, 30 grains daily. Three days later the patient commenced to talk; the choreic movements disappeared ten days after the commencement of treatment.

Case VI.—V. P. J., 11 years old. Slight chorea. Aspirin, 15 grains daily was given. The choreic movements disappeared at the end of ten days.

Case VII.—P. C., sister of the preceding; slight chorea. Aspirin, 15 grains daily. Cured after a number of days. Recurrence after a year; same treatment. Involuntary movements disappeared in the space of eight days.

Case VIII.—A. E., 12 years old. Marked chorea; standing erect impossible. Aspirin, 30 grains daily. Rapid improvment; twelve days later the choreic movements had entirely disappeared.

Case IX.—P. E., florist, aged 18 years. Extremely marked chorea. No signs of hysteria. The patient was unable to stand up. In bed she was so restless that each moment she threw the bedclothes to the ground. On November 2 she took aspirin, 45 grains. November 5, she was nearly free from the ataxic movements, except that the hands at times showed feeble involuntary contractions. November 14, the patient left the hospital.

Case X.—D. R., 6 years old; intense chorea, which was attended at its commencement with pains in the joints and mental disturbances. The little patient seemed to be idiotic and could not speak. Walking was almost impossible; the patient was also unable to keep herself in the upright position, the choreic movements being extremely intense. January 14 she received 15 grains of aspirin. The next day she was able to eat without assistance, and on the following day she talked well and walked about without aid. During the following days her condition remained stationary, the aspirin having been discontinued, but it was resumed five days later in doses of 30 grains daily. Three days afterward the choreic movements and the disturbance of speech had entirely disappeared.

Case XI.—D.V., domestic, aged 16 years; extreme chorea with inability to stand or walk about. The patient could not talk and alimentation was difficult on account of the trouble in swallowing. February 6, she received 45 grains of aspirin. Three days later she commenced to talk. The movements in the legs had ceased. She was able to hold herself up, but walking was still difficult. February 11, the discordant movements had entirely ceased, although the intellectual disturbances persisted and were even accentuated, the patient appearing to be idiotic. The aspirin was discontinued during six days, and then resumed in doses of 60 grains daily; but only a slow amelioration has since been demonstrated. Actually, however, the patient is on the road to recovery. She is able to eat well and is gaining in flesh.

The latter two observations show that if after taking aspirin for three or four days there is no distinct amelioration, or at least none of import, it is well to suspend the drug and to resume it at the end of several days in larger doses. It is advantageous to associate the use of aspirin with rest in bed, warm baths, liniments, and frictions with alcohol. The mode of life plays a very important part in the treatment of chorea, a milk diet being indicated in cases of anorexia; meats, and especially those finely hashed, fresh butter, eggs, and finally somatose, which has rendered us signal service in very rebellious cases, improve the general state of the patients, and thus promote recovery.

